# Mapping the knowledge domain of financial decision making: A scientometric and bibliometric study

**DOI:** 10.3389/fpsyg.2022.1006412

**Published:** 2022-10-19

**Authors:** Lin Guo, Junlong Cheng, Zhishuo Zhang

**Affiliations:** ^1^Business School, University of Newcastle, Newcastle upon Tyne, United Kingdom; ^2^Research Institute of Foreign Languages, Beijing Foreign Studies University, Beijing, China; ^3^School of Foreign Languages, Guizhou Education University, Guiyang, China; ^4^International Business School, Beijing Foreign Studies University, Beijing, China

**Keywords:** financial decision making, investment decision, hot spots, research trend, scientometric analysis, individual difference

## Abstract

Based on a 12-year bibliographic record collected from the Web of Science (Thomson Reuters) database, the present study aims to provide a macroscopic overview of the knowledge domain in financial decision making (FDM). A scientometric and bibliometric analysis was conducted on the literature published in the field from 2010 to 2021, using the CiteSpace software. The analysis focuses on the co-occurring categories, the geographic distributions, the vital references, the distribution of topics, as well as the research fronts and emerging trends of financial related decision making. The steady increase of papers published year by year demonstrated the increasing interest on this topic at the international level. The scientometric analysis of the literature showed that financial decision, investment decision, and financing decision stood out of the crowd of the research on FDM, suggesting their important role in FDM and its research. The results of citation burst analysis predicted the focus of topics, i.e., the impact of individual differences such as financial literacy, gender and age on FDM in the coming years. Different from the traditional approach of literature review, this bibliometric analysis offers a scientometric approach to reveal the *status quo* and the development trend of FDM by macro and quantitative means. In addition, future research directions for the field are recommended.

## Introduction

Financial decisions are one of the most significant, life-influencing decisions that individuals make, and they are made at various levels in the economic activities (Frydman and Camerer, 2016). Financial decision making (FDM) is gradually evolving into an independent construct from cognition and from financial management techniques (Lichtenberg et al., 2018), and it can be defined as a process of selecting an alternative from among a choice set concerning financial decision. The FDM process is multi-faceted and complicated, and is influenced by individual differences of the decision maker which would lead to distinct cognitive and neural mechanisms underlying the decision-making process. In addition, uncertainty about future income, social norms, and the complexity and diversity of financial instruments are a few of the factors that complicated these decisions (Lieber and Skimmyhorn, 2018). Consequently, the research on peoples’ financial decision has become the focus of intense interest among many scholars and professionals from a number of fields such as economy, business, management, psychology, neuroscience, and computer science (Bouzguenda, 2018). Nevertheless, there is an absence of comprehensive and quantitative review exclusively focused on FDM.

The purpose of our study is to provide a thorough overview of the *status quo* and the development of FDM research, and the prediction of its future tendency. Specifically, the work presented herein aims to identify and visualize the trends and the thematic patterns and topics of high interest to researchers in the domain of FDM, in the hope of helping researchers identify fundamental influences from categories, journals, references, research topics, and emerging trends in the field.

## Literature review

Financial decision can be classified into two categories, i.e., risk decision and intertemporal decision. Financial risk decision means that in a decision-making environment fraught with uncertainty, decision maker evaluates the cost and return and reaches a decision based upon the informed judgment of expected return and the determination of subjective probability (Hua and Wang, 2018). Intertemporal decision can be defined as the choices of financial resources made by an individual over a period of time, and it searches for an equilibrium between short-and-long term return (Frederick et al., 2002; Hua and Wang, 2018). Therefore, intertemporal decision refers to choices among outcomes which happen at different points in time.

Both financial risk decision and intertemporal decision making processes are affected by many factors and the past few decades have witnessed a surge of interest in the affecting factors of FDM. The relationship between financial decision and gender has been the subject of increasing interest for researchers and professionals. A large and growing body of behavioral and experimental evidence has been accumulated demonstrating significant gender differences in risk-taking and risk behavior in financial decision-making activities. Women exhibited more risk aversion and less risk tolerance than men when it came to financial decisions (Powell and Ansic, 1997; Croson and Gneezy, 2009; Charness and Gneezy, 2012; Gibson et al., 2013; Francis et al., 2015). Accumulating evidence has also indicated that women feel less confident about their ability in making financial decisions, although there is no clear evidence to suggest they are comparatively less capable of achieving desired target return (e.g., Estes and Hosseini, 1988; Stinerock et al., 1991; Zinkhan and Karande, 1991; Huang and Kisgen, 2013). It has also been documented that individuals’ decision-making capability changes with age. There is emerging literature on cognitive aging suggesting that older people have difficulty making cognitively demanding decisions (e.g., Mather and Carstensen, 2005; Tymula et al., 2013; Del Missier et al., 2017; Eberhardt et al., 2018). Although it has been well-documented that cognitive abilities declined with age and could harm older adults’ competence to make informed financial decisions (e.g., Gamble et al., 2015; Samanez-Larkin and Knutson, 2015), previous studies also observed that they possessed more experience-based knowledge that could facilitate their decision making in financial and business activities (e.g., Kovalchik et al., 2005; Bruine de Bruin et al., 2012; Eberhardt et al., 2018). The role of financial literacy, i.e., the ability to understand and effectively apply financial skills as well as personal financial information on FDM, has been explored, and the results showed that financial literacy can significantly affect the ability of decision makers to formulate strategies and have a competitive advantage in financial activities (e.g., James et al., 2012; Mouna and Anis, 2013).

Apart from the above-mentioned influential factors, prior work also indicates that individuals’ financial decision is influenced by many other factors such as emotion, emotional intelligence, behavioral biases and acute stress. It has been reported that emotions can modulate and modify FDM, because they can incur meaning to the available choices (e.g., Peters et al., 2007; Zaleskiewicz and Traczyk, 2020). With cognitively demanding tasks, individuals tend to rely more on their emotions than on deliberation to make financial decisions (e.g., Shiv and Fedorikhin, 1999). Emotional intelligence, the capacity to perceive, evaluate, comprehend and regulate emotions effectively in a context of emotional and intellectual development (Mayer and Salovey, 1997), has been suggested to impose a high potential influence on the managers’ funding decisions (Salovey and Mayer, 1990; Goleman, 1998). Behavioral bias is another factor that has been confirmed to impact the FDM process (e.g., Malmendier and Tate, 2005; Galasso and Simcoe, 2011). Under certain circumstances, financial decisions must be made under extraordinary stress, and previous studies have documented that suffering from stress can change an individual’s decision-making strategies and outcomes in financial and business activities (Starcke et al., 2008; Nofsinger et al., 2018).

Researchers have conducted bibliometric analysis of behavioral finance and behavioral economics *via* CiteSpace or VOSviewer. Costa et al. (2017) carried out a scientometric analysis on the relation between individuals’ behavioral finance and their financial/managerial decision making with cognitive biases. Employing CiteSpace software, they analyzed 889 papers published from 1990 to 2016, focusing on the most cited articles and authors, the authorship network, and the top journals in this field. The results indicated that the number of studies on the relation between cognitive biases, behavioral finances, and FDM has been increasing steadily throughout the years. They also found that cognitive biases such as overconfidence, confirmation and anchoring impacted the FDM process. Costa et al. (2018) presented a global overview of the domain of behavioral economics and behavioral finance in relation to individuals’ decision making *via* Citespace. The authors retrieved 2,617 articles from WoS database during the period of 1967−2015, indicating that the number of publications in this field has increased over time. The analysis revealed that behavioral economics covered subjects which relate individuals’ behavior with demand, price and consumption, as well as risk of investment and managerial making, and that behavioral finance mainly focused on the errors of judgment and the characteristics of financial related decision-making. Financial risk management is a vital part of human activity and all financial related decisions making involves risk management (Jirásková, 2017; Abdel-Basset et al., 2019). Ahmad et al. (2021) conducted a bibliometric analysis to explore the *status quo* and research trends based on the papers on financial risk management research indexed by WoS from 2004 to 2018. The analysis revealed the category distribution, the most influential articles, the top research areas, the prominent supportive funding agencies and the future research direction in the scientific field of financial risk management. López-Medina et al. (2022) presented a macroscopic overview of research on financial behavior in relation to individuals’ educational level, consumption, and money-saving, making contributions to the study concerning the sustainable development goals (SDGs). The data of 492 articles were retrieved from WoS during the time span of 1992 to 2021, and were visualized *via* VOSviewer software. The results of the analysis indicated a growing interest in this topic among the scientific community, and the major constructions of knowledge domain in financial education, consumption and savings decision making were visualized.

The overview of the literature showed that abundant research on FDM has explored the factors impacting FDM process, and that prior bibliometric studies have explored the relation between behavioral finance and financial/managerial decision making, but only focused on the effects of certain cognitive biases, like overconfidence, confirmation bias and anchoring effect (Costa et al., 2017). Other studies either explored the domain of behavioral finance in relation to individuals’ decision making (Costa et al., 2018) or the effects of financial literacy on savings and consumption decisions as well as investment decision making (López-Medina et al., 2022). In addition, the bibliometric analysis of financial risk management which can play a role in FDM was also conducted (Ahmad et al., 2021).These studies provided important insights into the further investigation of financial decision-making process, but the overall profile of the FDM research still awaits investigation.

Owing to the abundant literature on FDM, it is essential to classify the publications for the sake of identifying the foci of research, current hotspot issues, and further directions in this field as well as identifying the underlying reasons. However, the macro-profile of FDM research revealed by the extant literature is very limited and insufficient, and no research has yet been conducted to provide a macroscopic and quantitative overview of financial decision-related literature based on a bibliometric analysis of this topic. In view of this, this study used CiteSpace which was developed by Chen (2004), to map the knowledge domain of FDM research by examining the 2010−2021 bibliometric data in this field. Different from a traditional review of literature, a scientometric and visualization analysis of FDM literature can uncover another facet of the research frontiers on this topic by macro and quantitative means.

## Materials and methods

### The database selection

The initial stage of a comprehensive bibliometric analysis is the selection of the scientific database and the collection of the information about the published literature. The publications on the research of FDM are retrieved from the Thomson Reuters’ Web of Science (WoS) Core Collection, encompassing Social Sciences Citation Index (SSCI), Science Citation Index Expanded (SCI-EXPANDED), Arts and Humanities Citation Index (A&HCI), Conference Proceedings Citation Index-Science (CPCI-S), Conference Proceedings Citation Index-Social Science and Humanities (CPCI-SSH). The WoS database is one of the most prominent databases in the scientific realm for indexing publications and among the most commonly used bibliographic data sources by researchers, allowing access to thousands of journals from all over the world and from eminent international publishers (c.f., Cicea et al., 2021). WoS is among the most reliable, global and independent citation databases which can fulfill the requirement of bibliometric analysis (Ahmad et al., 2021). In short, WoS was selected due to its (i) prestige in academia (Yang et al., 2022); (ii) a wide range of related journal articles on FDM research. Moreover, selecting just one database can allow the standardization and consistency of data (Prado et al., 2016). Hence, the present study chose WoS as the data source for our data preparation.

### Determination of search subject and time span

The second stage involves the determination of the search subject and time span. In this study, TS = (“financ* decision” OR “financ* decision making” OR “financ* decision-making”), in which “TS” means topic (title, abstract, and keywords), and the symbol “*” broadens the search for all words which start with the retrieved term, despite their complement (*finance*, *financial* and *financing*). The data retrieval was executed on May 31st, 2022.

The present study focused on the bibliometric analysis of the literature published from 2010 to 2021, disclosing the *status quo* and recent development of FDM research. Publications prior to 2010 are excluded, yet the results would not be undermined by the exclusion, as only a few relevant publications can be identified in that period. The document type was article or review^1^, and the language of the retrieved literature was in English. Having excluded non-English and publications irrelevant to FDM or beyond the research scope of this study, a total number of 870 papers were selected as the sample for the subsequent analyses.

### Selection of software

The third stage involves the identification of the proper software for the analysis. In this study, CiteSpace was employed to study the literature of FDM to reveal the research patterns, hot topics in the domain of FDM and explore the possible underlying reasons for the development of the field, focusing on the most productive authors, category co-citation, document co-citation, journal co-citation and citation bursts. Developed by Chen (2004), CiteSpace was a widely employed tool for visual exploration of literature in a scientific community. CiteSpace can identify the fast-developing topic areas; detect citation bursts in the assemblage of literature; disassemble a network into clusters, and label clusters automatically with terms from citing publications, geospatial patterns of cooperation and areas of collaboration at an international level (Chen, 2014). It supports temporal and structural analyses of a wide range of networks obtained from scientific literature such as author co-citation networks, document co-citation networks and collaboration networks, and also supports networks of hybrid node types such as terms, institution, countries, and hybrid link types including co-occurrence, co-citation, as well as directed citing links (Chen, 2004). In addition, CiteSpace can produce more distinct and detailed images than VOSviewer; and it adopts various algorithms (e.g., LLR, LSI, and MI) to extract information for cluster interpretations, and generates different tags for scholars to choose from.

### Data analysis

**FIGURE 1:**
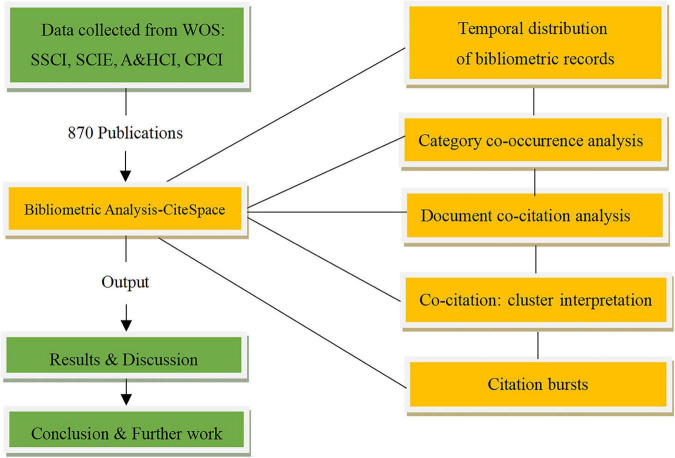
Processes of financial decision-making bibliometric records analysis.

The fourth stage is concerned with the data analysis. The following analysis is performed for data analysis: temporal trend of publications, the vital journals, geographical distribution, category co-occurrence, document co-citation, and citation bursts. The research process involved in this study is depicted in Figure 1, which was from the present authors’ own conception.

## Results

All the tables and figures in this section were from the authors’ own conception *via* on Microsoft Excel and Citespace software.

### Temporal distribution of bibliometric records and top journals

**FIGURE 2:**
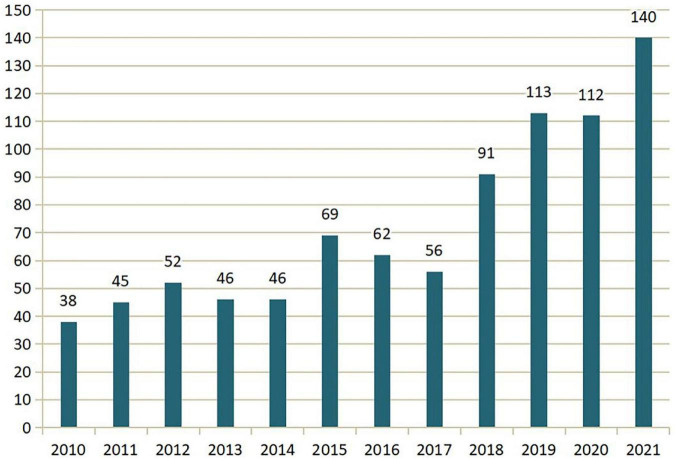
Numbers of published articles (2010–2021).

We retrieved 870 publications in the field of FDM, and Figure 2 displays the annual number of published works for each year. As is shown in Figure 2, there was a steady increase in the number of published works in FDM research from 2010 to 2021. This indicates an increasing and sustained interest from researchers in this topic over the past 12 years.

The retrieved articles and reviews on FDM were carried in 451 journals of a variety of disciplines, which suggested that the studies on this topic have attracted attention of researchers from various research fields. The top ten most fruitful journals were shown in Table 1. *Journal of Marketing Research* ranked the first in the number of published papers (19), followed by *Frontiers in Psychology* (15) and *PLoS One* (15), and these journals belong to the disciplines of marketing, business, economics, psychology and multidisciplinary science.

**TABLE 1:** Top ten most fruitful journals.

No.	The name of journals	The number of published papers
1	Journal of Marketing Research	19
2	Frontiers in Psychology	15
3	PLoS One	15
4	Expert Systems with Applications	14
5	Journal of Consumer Affair	12
6	Journal of Economic Behavior Organization	12
7	Journal of Economic Psychology	12
8	Decision Support Systems	11
9	Journal of Behavioral Finance	11
10	Journal of Consumer Research	11

### Geographical distribution

Based on the address(es) of the author(s) listed in the retrieved data, it was observed that publications in FDM originated from 74 different countries and regions. The influence of a country in the analyzed research area can be determined by its number of publications and the citations per document in the collection (Dzikowski, 2018), as listed in Table 2, where the top 10 productive countries/regions were shown. It is noted that United States is the most prolific country in FDM research (337 documents), accounting for 38.7% of the total publications. The citation of per document of United States was also the highest (8,834 citations). China ranked second in the number of publications (112) and citation counts (2,038). The subsequent productive countries are England, German and Australia, with 89, 64, and 58 documents, respectively, which were much less than those of United States and China.

**TABLE 2:** The top 10 prolific countries/regions in financial decision-making research.

Rank	Countries/Regions	Continent	Documents	Citations	Avg. pub. year	Avg. citations
1	United States	North America	337	8,834	2016.41	26.21
2	China	Asia	112	2,038	2018.08	18.20
3	England	Europe	89	1,584	2016.58	17.80
4	Germany	Europe	64	1,321	2017.13	20.64
5	Australia	Oceania	58	621	2017.29	10.71
6	Canada	North America	34	628	2016.44	18.47
7	Netherlands	Europe	30	1,121	2016.83	37.37
8	Switzerland	Europe	23	475	2017.09	20.65
9	France	Europe	22	612	2016.23	27.82
10	Italy	Europe	20	286	2016.80	14.30

**FIGURE 3:**
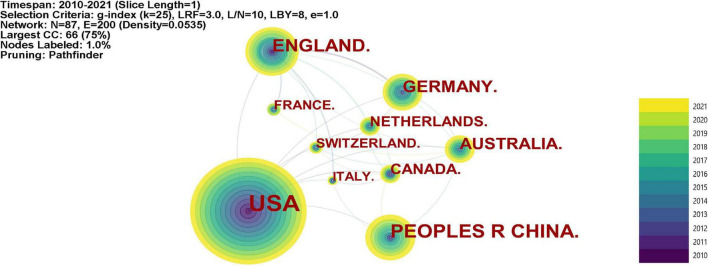
Countries/regions collaboration network of financial decision making research.

Figure 3 visualized the co-authorship between different countries and regions in the research domain of FDM. The amount of publication is represented by the size of circle, and the thickness of links indicates the strength of collaboration (Li and Liu, 2020). Generally, cooperative countries are most likely to center around the most prolific countries in publications and are correlated geographically (Zheng et al., 2016). As the most prolific country in FDM research, United States has played a significant role in this research field *via* a comprehensive collaboration with other countries/regions, such as China, England, Germany, Canada etc. Although China ranked the second in its publication in this field, it has cooperated less with other countries/regions. China only collaborated with United States, Canada, Australia and Italy, and had lower citations per document compared with other prolific countries/regions. This may be partly attributed to the fact that China, as a country in Asia, is geographically distant with other countries/regions. In addition, owing to England’s and Australia’s comprehensive cooperation with other countries/regions in this topic, there were major collaboration clusters gathering around England and Australia.

### Category co-occurrence analysis

**FIGURE 4:**
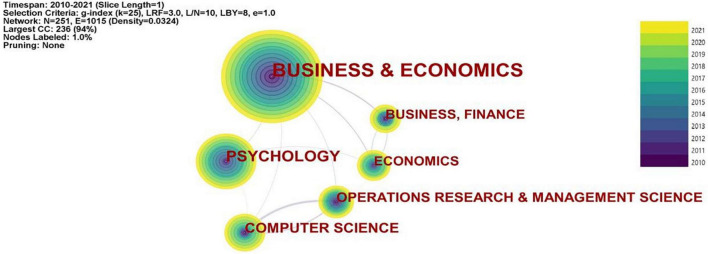
The most highly co-occurring categories.

Category analysis can reflect the distribution structure of the research relevant to a subject matter across various disciplines in the scientific community. Figure 4 visualizes the distribution of financial decision-making research across different disciplines and presents the top 6 co-occurring categories in FDM from 2010 to 2021. Nodes in the network here are cited references, and lines between nodes are co-citation links. Business and Economics, Psychology, Computer Science, and Operational Research and Management Science are the top four disciplines, indicating their close relationship with the topic. It should be noted that the study of FDM is of particular concern for researchers in the fields of operational research and management science as well as computer science, suggesting the combined attention to FDM research from both social science and natural science.

Table 3 illustrates the most highly co-occurring categories. The top ranked category is Business and Economics (citations = 386), and the fifth category is Economics (citations = 58), and the sixth is Business and Finance (citations = 57). These categories fall into the traditional disciplines of business, economics and finance, with a total citation of 501 (386 + 58 + 57), indicating financial decision research attracts most attention among researchers from these disciplines (e.g., Cesarini et al., 2010; Hildebrandt and Knoke, 2011; Van Rooij et al., 2011; Charness and Gneezy, 2012; Kolm et al., 2014). The second category is Psychology (citations = 118), which demonstrates that the psychological mechanisms of financial decision receive great attention from scholars over the past two decades (e.g., Carr and Steele, 2010; Serido et al., 2013; Brown and Taylor, 2014; Frydman and Camerer, 2016). The third category is Computer Science (citations = 76), indicating researchers explore financial decision-making process from the perspective of the computer science and technology (e.g., Merigó and Casanovas, 2011; Xu and Xia, 2011; Göçken et al., 2016; Zhang et al., 2021). The fourth category is Operational Research and Management Science (citations = 61), which falls into the discipline of management, indicating financial decision research attracts great interest from researchers in this discipline (e.g., Zopounidis et al., 2015; Kraus and Feuerriegel, 2018; Zhu et al., 2019).

**TABLE 3:** The most highly co-occurring categories.

Rank	Citation counts	References
1	386	Business and Economics
2	118	Psychology
3	76	Computer Science
4	61	Operations Research and Management Science
5	58	Economics
6	57	Business, Finance

To sum up, according to the results of category analysis, FDM research in the past 12 years can be roughly classified into two broad categories: the research related to traditional disciplines such as business, economics, and management, and the newly multidisciplinary research relevant to psychology and computer science, which plays an increasingly important role in FMD research these years.

### Document co-citation analysis

**FIGURE 5:**
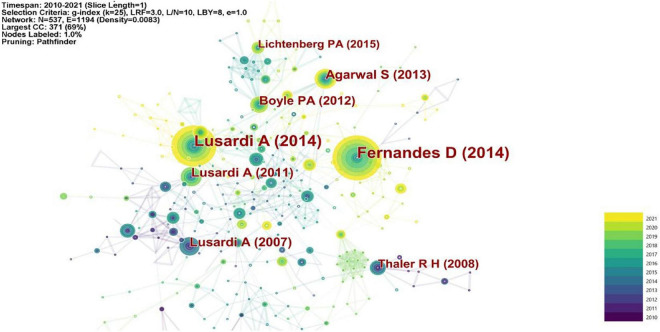
Key articles in financial decision making.

The 870 bibliographic records published between 2010 and 2021 were visualized and a 1-year time slice was selected for analysis, generating the document co-citation network as illustrated in Figure 5. There were 537 individual nodes and 1,194 links, representing cited articles and co-citation relationships among the whole dataset respectively.

The most cited article is published by Fernandes et al. (2014). They performed a meta-analysis of the relationship among financial education, financial literacy, and financial behaviors, and observed that interventions to improve participants’ financial literacy had little effect on the improvement of their financial behaviors (accounted for only 0.1% of the variance), and that weaker average effects were observed for low-income participants. Based on the results of their meta-analysis, Fernandes et al. (2014) also concluded that studies on financial education so far have noticeable limitations which had been undermined by the presence of seemingly larger impacts in correlational studies.

The second most cited article is by Lusardi and Mitchell (2014) who undertook an assessment on financial literacy, a rapidly growing subject of economic research. They made use of the results of previous surveys to identify the subgroups of the population with the least financial literacy, and examined how financial literacy influenced economic decision-making in the United States and other countries. The work done by Agarwal and Mazumder (2013) is the third most cited one. They analyzed two examples where suboptimal behavior was well defined, and which were used to explore the impacts of cognitive abilities on consumer financial decisions. The first example involved the credit cards’ optimal use after balance transfers for convenience use; the second one featured a financial fault on the application of a home equity loan. They found that consumers with higher overall test scores and math scores made fewer financial mistakes. The fourth most cited article is Lusardi and Mitchell (2011). According to their international research, they found that women had less financial literacy than men, and that the middle-aged group had more financial literacy than the young and older people, and that more education enabled individuals to be more financial literate. More importantly, instrument variables (IV) estimates showed the effect of financial literacy on retirement planning was underestimated.

The fifth most cited article, authored by Lusardi and Mitchell (2007), focused on Baby Boomers who were on the edge of retirement, indicating there was a strong positive correlation among wealth levels, retirement planning and economic literacy. They also found that distribution of total value of assets among these Early Baby Boomers was very unbalanced, and that lots of Americans on the verge of retirement had very little accumulated wealth outside their homes (Lusardi and Mitchell, 2007).

### Co-citation: Cluster interpretation

**FIGURE 6:**
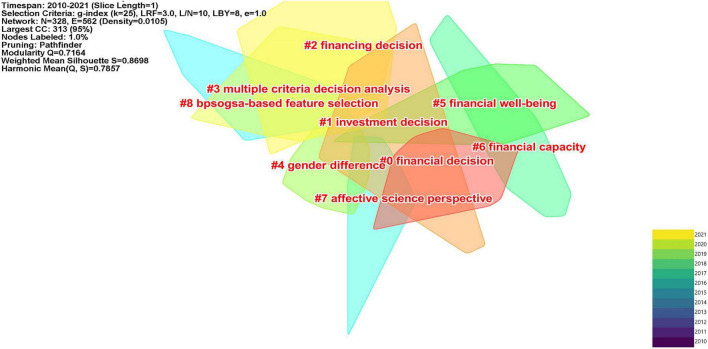
Co-citation clusters (2010–2021).

**FIGURE 7:**
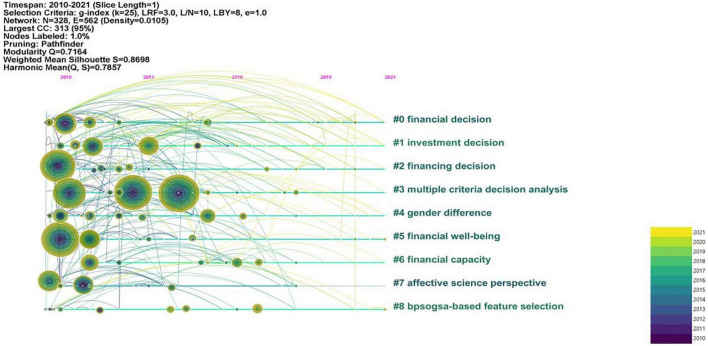
Timeline view of the financial decision-making knowledge domain.

The 870 bibliographic records generated 15 clusters in total. Among these clusters, cluster #0, cluster #1, cluster #2, and cluster #3 present the highest co-citation, indicating that these four clusters are the critical and active study efforts in the duration of 2010−2021 in the domain of FDM. Figures 6, 7 present the 9 main clusters in different layouts.

The largest cluster (#0), labeled as financial decision, included 43 articles and had a silhouette value of 0.902, with more attention being focused on financial decision-making ability of groups of individuals with different cognitive impairments. The top five most cited articles are Tsai and Young (2010), Willner et al. (2010), Sunderaraman et al. (2019), Bangma et al. (2020), Yu et al. (2021). Willner et al. (2010) adopt a more realistic task of financial decision making to replicate and extend the previous results, in order to test whether participants with intellectual disabilities (ID) could “weigh-up information.” They observed that “weighing up” information was not evident for people with ID. Tsai and Young (2010) investigated how anger and fear affect the escalation of commitment in a situation of FDM. Study 1 demonstrated that the fear was associated with less escalation of commitment than anger in a personnel recruitment appraisal. Study 2 replicated the pattern of results of Study 1. Additionally, angry individuals recognized lower risk in their initial financial decisions than the fearful individuals, which resulted in the increasing tendency of escalating commitment. The current findings indicated that negative emotions might not always alleviate the escalation of commitment. Yu et al. (2021) tested whether metamemory deficit could lead normal older people to make poor financial decisions. They found that metamemory deficit impaired older adults’ FDM. Sunderaraman et al. (2019) examined how the financial capacity was impacted by chronic, moderate to severe acquired brain injury (CABI). Both healthy and CABI participants completed Financial Competence Assessment Inventory (FCAI) and a neuropsychological battery. The results showed that CABI group had lower performance on each dimension of FCAI, suggesting that CABI can impair the financial capacity. Bangma et al. (2020) investigated the negative effects of attention deficit hyperactivity disorder (ADHD) on impulsive buying and the use of financial decision styles. It is safe to conclude from the results that adults with ADHD intended to buy more impulsively, and use avoidant and spontaneous financial decision styles more frequently.

Cluster #1 is labeled as investment decision, which focused on the factors that may impact the investment decisions made by individuals or households. There are totally 34 articles in this cluster and with a silhouette value of 0.782. The three most cited papers are Petersen et al. (2015), Kim et al. (2016), Gutsche et al. (2021). Kim et al. (2016) found that the inertia patterns displayed in investment management over the life cycle of many households can be explained by the “calibrated dynamic life cycle portfolio choice model” they have proposed. They also concluded that investors were occupied when managing money, so they had to give up acquiring job-specific skills, and their efficiency in FDM changed with age. Gutsche et al. (2021) empirically examined decision-makers’ individual sustainable investment behaviors in Japanese households from the following perspectives: (1) the awareness of sustainable investments of individual investors, (2) the present share of sustainable investments in their portfolios, (3) their future intention to invest sustainably. The results indicated that individual investors considered financial literacy, signals of society or word-of-mouth learning, financial performance perceived, and preferences of risk as the vital determinants for current sustainable investment. In addition, the future decision of sustainable investment was driven by “individual environmental values and ecological political identification” (Gutsche et al., 2021). Petersen et al. (2015) empirically tested both the direct impact of national culture on FDM and its moderating impacts on the link between a business’ marketing efforts and the FDM of its clients. They found that national culture directly affected consumer’s FDM, and the financial service companies could moderate the impact of marketing efforts, which indicates that such firms should take national culture into account of customers management.

Cluster #2 is labeled as financing decision and it included 36 articles and is with a silhouette value of 0.855. The top three most cited papers are Wang (2010), Hernádi and Ormos (2012), Munir and Li (2016). Wang (2010) made a comparison on the financial strategies adopted for the high-tech companies in Chinese Mainland and Taiwan through path analysis and directed graph model. Their results suggest that the financial strategies of high-tech firms’ across the Strait have various casual structures and their financial decisions have an influence on their company’s performance, capital expenditures and so on. Munir and Li (2016) attempted to explore the non-linearity relationship between the power of chief executive officer (CEO) in small and medium sized companies leverage by employing a threshold estimation technique. The results revealed that the relationship between the power of CEO and value-based leverage is an inverted U-shaped one, suggesting that CEOs with higher ability are inclined to use lower leveraged in financial decision environment. What is more, Hernádi and Ormos (2012) examined the factors influencing small and medium-size enterprises’ (SMEs) capital structure and their choices, and verified the relevance of three major theories which have been constructed mainly for developed markets. They came to the conclusion that the financial decision-making procedure of most firms in Central and Eastern Europe (CEE) countries are consistent with that of the developed countries.

Cluster #3 is labeled as multiple criteria decision analysis and with a silhouette value of 0.951, which meant that researchers concentrated on the multiple potential factors that could account for the financial decision-making process. There are totally 57 papers in this cluster. The three most cited articles are Kaufmann et al. (2012), Johnston et al. (2016), Ranganathan (2021). Ranganathan (2021) investigated the role of individuals’ risk attitude in FDM. The empirical results showed that the personal focus can encourage individuals to take more risks in decision making as compared with the social focus. This research makes contribution to the exploration on the psychometric structure of risk in the field of FDM. Johnston et al. (2016) explored the effects of the partners’ respondent, health, cognitive ability, and personality on FDM in the household. The results indicated that: (1) mental health played a more important role in accounting for the allocation of household FDM relative to physical health; (2) high cognitive ability was related to a high likelihood of decision-making responsibility; (3) personality could significantly predict who was the decision-maker in the household, and great effects were observed for conscientiousness and agreeableness. Meanwhile, the authors found that many results were sensitive to whether the decision-maker is the male or female, suggesting the role of gender in household FDM. Kaufmann et al. (2012) investigated the effects of debiasing measures and corresponding contextual variables on the effectiveness of financial decision in the supplier selection process. The results indicated individual environmental factors and organizational factors exerted different effects on the employment of debiasing methods for supplier selection decisions. In addition, debiasing tactics were found to have different impacts on the financial effectiveness of supplier selection decisions, with either a positive or negative effect.

Cluster #4 is labeled as gender difference, which means that the investigation of the effects of gender on FDM have attracted researchers’ attention. This cluster includes 28 articles and the top three most cited papers are Yordanova and Alexandrova-Boshnakova (2011), Nelson (2015), Harrison et al. (2020). Yordanova and Alexandrova-Boshnakova (2011) administered a survey to 382 Bulgarian owner-managers of business to explore the gender differences in these businesses owners’ decision making under risk. Their findings suggest that women business owners have a lower tendency toward risk in decision making because of their risk preference, age, and outcome. However, Nelson (2015) believed the conclusion that “women are more risk averse” merited more investigation. The review revealed that empirical evidence was not sufficient in supporting such conclusion and that the cultural and contextual influences also contribute to the gender differences in risk-taking behavior. Harrison et al. (2020) also found that this kind of stereotype threat exerts negative influences on women investors, depressing their investment performance.

Other clusters, such as Cluster #5 (financial wellbeing), Cluster #6 (financial capacity), Cluster #7 (affective science perspective), and Cluster #8 (bpsogsa-based feature selection) are also worthy to be mentioned. Cluster #5 mainly focuses on how people’s feeling might affect their financial participation. Related studies suggest that the accumulation of financial resources boost one’s confidence, and feeling powerful enables individuals to save more (Garbinsky et al., 2014). Meanwhile, pleasant feeling was found to facilitate household’s behavior in risky financial market (Cui and Cho, 2019). Cluster #6 is labeled as financial capacity, which is an important determinant in the study of financial exploitation. Financial Decisional Abilities Model proposed by Lichtenberg (2016) illustrated the contextual, intellectual, and psychological factors that might influence people’s FDM. Besides, six clinical models presented by Marson (2016) were of clinical significance in that they provided conceptual guidance for a sound clinical and forensic assessment concerning financial capacity. Prior studies also showed that individuals with moderate to severe traumatic brain injury impaired financial capacity (e.g., Dreer et al., 2012). Researchers exploring the relationship between brain aging and financial decision-making changes can be found in Cluster #7. Older adults may be confronted with the challenges of FDM as their brain-related processing changes (Weierich et al., 2011). It was observed that individuals’ priori regions of the bilateral nucleus accumbens (NAcc) and right anterior insula were the neural substrates of social decision-making in financial choice tasks (Cartmell et al., 2014). Participants’ anterior cingulate cortex (ACC) and superior frontal gyrus (SFG) were also found to be activated when they choose to disobey the financial authority in an investment game (Suen et al., 2014). The term label of Cluster #8 suggested that some credit scoring models and classification methods can be used to improve the individuals’ performance in financial decision-making activities (e.g., Gorzałczany and Rudziński, 2016; Edla et al., 2018; Wang et al., 2021).

In sum, the resulting clusters represent the main research focus in the knowledge domain of FDM. The interpretation of clusters demonstrates that the topic “financial literacy” is the most active area of research, focusing on the role (also interventions) of financial literacy in individual’s financial decision-making performance.

### Citation bursts since 2016

Citation bursts can be applied to exploring the research trends of a certain research filed (Chen, 2006; Hou and Bo, 2016; Wang et al., 2019), and latest ongoing bursts can disclose the future trends to some extent (Guo, 2017; Wang et al., 2019). All citation bursts since 2016 were chosen to be analyzed in order to reveal the emerging trends of FDM research; 2016 was set as the year point since all ongoing citation bursts appeared after this year. Table 4 displays the recent publications with citation bursts with references from 2016 onward. By analyzing the sudden increase in the citations of specific references, researchers can discover the research interests at present, and the research trends of a certain knowledge filed in the future (Wang et al., 2019). In this section, we will provide a summary of the main future trends based on the latest citation burst presented in Table 4, which displays the references in a way of strength of citation bursts and the beginning year of the burst. In total, 15 papers with the end year of 2021 are classified to represent different emerging trends in the future.

**TABLE 4:** References with the most recent citation burst since 2016.

References	Strength	Begin	End	2016–2021
Boyle et al., 2012	2.86	2016	2021	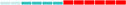
Charness and Gneezy, 2012	3.19	2018	2021	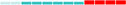
Lusardi and Tufano, 2015	2.83	2018	2021	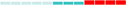
Fernandes et al., 2014	2.41	2018	2019	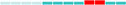
Beshears et al., 2015	1.81	2018	2021	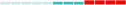
Bhattacharya et al., 2012	1.77	2018	2021	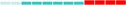
Hadar et al., 2013	1.77	2018	2021	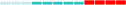
Gathergood, 2012	1.77	2018	2021	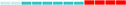
Lichtenberg et al., 2015	2.33	2019	2021	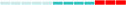
Lusardi and Mitchell, 2014	2.04	2019	2021	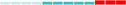
Yan et al., 2016	2.02	2019	2021	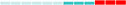
Bangma et al., 2017	2.02	2019	2021	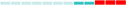
Fonseca et al., 2012	1.85	2019	2021	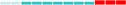
Behrman et al., 2012	1.85	2019	2021	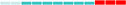
Hastings et al., 2013	1.6	2019	2021	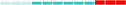

The most compelling trend is the influence of financial or debt literacy on individual’s FDM. Lusardi and Tufano (2015) found that people with lower debt literacy were more likely to transact in high-cost ways and use high-cost borrowing. They were reported to have excessive debt loans, and were incapable to judge their debt position. Fernandes et al. (2014) and Lusardi and Mitchell (2014) are also the first and second most cited articles revealed in the analysis of the most cited articles. This unsurprising coincidence suggests that these two studies have ideally followed the research trend of FDM. Behrman et al. (2012) explored how American household wealth accumulation was affected by financial literacy. They found that improved financial literacy could help families to accumulate wealth much better. Hadar et al. (2013) concluded that if the increase of consumers’ objective knowledge (OK) regarding financial instruments diminished subjective knowledge (SK), their willingness to invest would be deterred. The results of their 4 experiments in which they used different SK manipulations showed that: (1) high SK led to an increase in consumers’ willingness to select a risky investment; (2) enhancing consumers’ willingness to enroll in a retirement program resulted from asking them easy financial questions; (3) SK was diminished if a mutual fund’s information was explained technically, and decreased the selection of the fund; (4) a salient missing information could result in a decrease in investment, holding the constant objective information. Gathergood (2012) found that lack of self-control and financial literacy were the main causes of non-payment of consumer credit and excessive debt. Also, lacking self-control played a stronger role than that of financial literacy in explaining the over-indebtedness. Hastings et al. (2013) mainly discussed whether the public of United States were aware of the effects of financial education and financial literacy on economic outcomes.

The second category discusses how gender differences impact decision-makers’ FDM. Charness and Gneezy (2012) set up a simple investment game for participants. They found that women invested less, and were more risk averse than men. Fonseca et al. (2012) found that gender differences in financial literacy could be explained by the coefficient differences, or how literacy was formed. This can help government to develop proper policies to reduce the gender gap, and improve the saving and investment decisions of females.

The third direction is to explore the aging effect on individuals’ FDM. Boyle et al. (2012) suggested that even older adults without Alzheimer’s disease (AD) or mild cognitive impairment can lead to poor decision making, as a result of cognitive decline. Bangma et al. (2017) also found that normal aging had a negative impact on a complex financial decision-making task. Lichtenberg et al. (2015) constructed a new assessment instrument called the Lichtenberg Financial Decision Rating Scale (LFDRS), so as to evaluate whether older people’s financial decision was an autonomous, capable choice.

The study conducted by Bhattacharya et al. (2012) is also worth being mentioned. They recorded what happened when active retail customers were offered unbiased investment advice. The results showed that only about 5% of investors could obtain the unbiased information, and that their portfolio efficiency was not improved significantly. This indicated that the mere accessibility of unbiased financial information did not sufficiently benefit retail investors.

Overall, the emerging research trend identified from the latest citation bursts since 2016 suggests that the role of financial literacy, gender difference and age in FDM will still be appealing to researchers in the domain of financial related decision making in the upcoming years.

## Discussion

Based on the bibliographic record collected from the database, the present study attempted to provide a macroscopic overview of the knowledge domain of financial related decision making. In general, there has been a steady increase in FDM research from 2010 to 2021, and the annual number of articles published on FDM increased from 38 to 140, suggesting an increasing and sustained interest from researchers on this topic. It is reasonable to conclude that the researchers will continue to pay close attention to the research domain of FDM in the subsequent decades. The analysis of top journals identified that the most prolific journals are *Journal of Marketing Research*, *Frontiers in Psychology* and *PLoS One*. These journals and other productive journals belong to a number of disciplines such as marketing, economics, business, psychology and multidisciplinary science, indicating the interdisciplinary nature of the FDM research.

The results of geographical distribution indicated that the United States, China, and England are the most prolific country in FDM research, and there was a major collaboration cluster gathering around United States, suggesting its significant role in FDM research through its close cooperation with other countries/regions. China ranked second in the number of publications, but it had less cooperation with other countries/regions. It might because that China is geographically far apart from other countries/regions. In the future, scholars in China are recommended to cooperate more with scholars from other countries and continents, performing collaborative research in FDM. The results of co-occurring category analysis revealed that financial decision-making research is of most concern in the disciplines of Business and Economics, Psychology, Computer Science, which demonstrated this topic was closely related to these disciplines.

The results of the co-citation analysis revealed a number of prominent clusters, such as financial decision, investment decision, financing decision, multiple criteria decision analysis, gender difference, affective science perspective and so on. Financial decision, investment decision and financing decision are the top three clusters in the field of FDM, suggesting their significant role in this research domain. Financial decisions are of vital importance both for individuals and companies. A company’s performance is closely related to the financial capacity of their leaders (Wang, 2010; Hernádi and Ormos, 2012; Munir and Li, 2016). However, studies suggest that different cognitive impairments contribute to some groups’ poor performance in financial related decision making. Individuals with intellectual disabilities (Willner et al., 2010), metamemory deficit (Yu et al., 2021), acquired brain injury (Sunderaraman et al., 2019), and attention deficit hyperactivity disorder (Bangma et al., 2020) impair their financial capacity.

Prior studies have also investigated the effects of individual differences on FDM. Factors documented to have an impact on the FDM processes and/or outcomes are age (Weierich et al., 2011), gender (Yordanova and Alexandrova-Boshnakova, 2011; Nelson, 2015; Harrison et al., 2020), risk attitudes (Ranganathan, 2021), feelings (Tsai and Young, 2010; Garbinsky et al., 2014; Cui and Cho, 2019), health and personality (Johnston et al., 2016), households’ choice portfolios (Kim et al., 2016), investment awareness and intention (Gutsche et al., 2021), environmental and organizational factors (Kaufmann et al., 2012), as well as national culture (Petersen et al., 2015).

Neural evidence of individual differences on FDM process has also been documented. Using functional Magnetic Resonance Imaging (fMRI) techniques, Cartmell et al. (2014) found that individuals’ priori regions of the bilateral NAcc and right anterior insula were involved in social decision-making tasks. What is more, Suen et al. (2014) employed fMRI to examine the differences in brain activation when participants received expert and peer investment advice and found that there was an increased activation of participants’ ACC and SFG when they chose to disobey expert advice in an investment game.

## Conclusion and further work

In the current study, a bibliometric analysis of the published literature in the field of FDM was conducted. CiteSpace software was used to quantitatively and visually review the literature. The information of the temporal distribution, co-occurring categories, journals, references of the collected bibliometric records, and research trends are examined. The steady increase of papers published year by year demonstrates the increasing interest in this topic at the international level. The scientometric analysis of the literature show that financial decision, investment decision, financing decision, multiple criteria decision analysis and gender difference stand out of the crowd of FDM research, and the effects of financial literacy, gender difference and age on FDM represent the research fronts for this topic, which contributes to the understanding of the knowledge domain the hot topics of this field and research trends visually and efficiently. Therefore, the current study contributes to providing a quantitative way for identifying the research focus and predicting the emerging trends of the FDM research.

Nevertheless, due to the interdisciplinary nature of the research on FDM, further efforts are required so as to provide a comprehensive profile of the mechanisms underlying financial decision-making behavior. For further studies, three future directions are worth considering: (1) investigating the interactive effect between individual differences and text variables on FDM process; (2) using online method to examine the real-time processes of FDM; (3) innovating research methods such as the combination of Event-related potentials (ERPs) and fMRI to explore the neural mechanisms underlying financial decision-making process.

## Data availability statement

The original contributions presented in this study are included in the article/supplementary material, further inquiries can be directed to the corresponding author/s.

## Author contributions

LG contributed to the conceptualization, data collection, formal analysis, methodology, visualization, writing – original draft, review, and editing the manuscript. JLC collected and visualized the data and wrote, reviewed, and edited the manuscript. ZSZ wrote and edited the manuscript. All authors contributed to the article and approved the submitted manuscript.

## Footnotes

1 In this article, the review articles do not include book reviews.
